# A survey of vocal mimicry in companion parrots

**DOI:** 10.1038/s41598-022-24335-x

**Published:** 2022-12-05

**Authors:** Lauryn Benedict, Alexandra Charles, Amirah Brockington, Christine R Dahlin

**Affiliations:** 1 Department of Biological Sciences, University of Northern Colorado, Greeley, CO 80639 USA; 2 Department of Biology, University of Pittsburgh at Johnstown, Johnstown, PA 15904 USA

**Keywords:** Behavioural ecology, Social evolution, Learning and memory, Animal behaviour

## Abstract

Parrots are one of the rare animal taxa with life-long vocal learning. Parrot vocal repertoires are difficult to study in the wild, but companion parrots offer a valuable data source. We surveyed the public about mimicry repertoires in companion parrots to determine whether vocal learning varied by (1) species, (2) sex, (3) age, and (4) social interaction with other parrots. Species differed significantly in mimicry ability, with grey parrots (*Psittacus erithacus*) having the largest mimicry repertoires. Analyses of all birds (n = 877) found no overarching effects of sex, age, or parrot-parrot social interactions on mimicry repertoires. Follow up analyses (n = 671), however, revealed a human bias to assume that talking parrots are male, and indicated that five of the 19 best-sampled species exhibited sex differences. Age-specific analyses of grey parrots (n = 187) indicated that repertoire size did not increase during adulthood. Most parrots were capable of improvisation (e.g. rearranging words) and used mimicry in appropriate human contexts. Results indicate that parrot vocal production learning varies among and within species, suggesting that the mechanisms and functions of learning also vary. Our data provide a rich foundation for future comparative research on avian vocalizations, and broaden our understanding of the underpinnings of communicative behavior and learning across all animals.

## Introduction

True language is unique to humans but multiple animal lineages show vocal abilities that include critical elements of language-like signaling^1,2^. In particular, there is much interest in understanding when and how animals can learn to imitate a variety of sounds, and reproduce them in appropriate contexts^3^. Four lineages of mammals and three lineages of birds are known to be vocal production learners; these groups provide fertile testing grounds for studies of the factors shaping vocal signaling^4,5^. Both mammals and birds in those lineages show learning early in life as vocal repertoires develop^4,6^. Oscine passerine birds, hummingbirds, bats and other groups learn new vocalizations from conspecifics as social environments change with individuals encountering new communication partners^4,7,8^. Cetaceans, pinnipeds, and elephants can imitate the sounds of conspecifics, and can also learn to imitate human-generated sounds^4^. The groups that most closely imitate human sounds, however, are birds—in particular, many species of parrots are outstanding human mimics^9^.

Researchers studying vocal learning in birds tend to focus on songbirds as model systems. Extensive research on songbirds has revealed (1) that bird species have differently sized repertoires of song types and syllable types that some species can recombine within songs, (2) that male and female birds often show very different song repertoires, and (3) that patterns of learning vary with age or social context^6^. Parrot vocal repertoires have been studied far less extensively than songbirds, with some exceptions^10–14^. This is due in part to the logistical challenges of tracking parrots in the wild^15^. Parrots, however, present an exciting system for the study of animal vocal repertoires, as they have extensive learning capabilities and unique body and brain structures hypothesized to be analogous with human physiology that supports vocal learning^16–18^.

Age, sex, species identity, and social setting all have the potential to shape parrot vocal repertoires. Parrots can learn new sounds relatively quickly, but can also continue to learn new sounds over their entire life spans^15,19^. In general, male and female parrots are thought by scientists to vocalize equally, but there are some species that show sex-differences^15^. For example, budgerigar (*Melopsittacus undulatus*) males are more likely than females to use a courtship song, and to adjust their vocalizations to match those of their mates^20,21^. Most parrot vocalizations, however, are not used primarily in courtship, but function instead in social communication within flocks, often mediating nest defense and foraging^15^. Such social interactions are critical to parrot communication, and some authors have hypothesized that social interactions may drive vocal repertoire complexity^22,23^. Complete natural vocal repertoires have only been calculated for a few species of parrots, which show a wide range of repertoire sizes, from 15 to over 100 note types^24,25^. Popular wisdom often claims that grey parrots (*Psittacus erithacus*) are the best vocal learners^26^. Grey parrots do show many advanced cognitive abilities, but the only study of wild birds suggests that they have large, but not huge, repertoires of about 39 vocalization types^17,27,28^. Overall, research on wild parrots confirms that species have differently sized repertoires, but much remains to be learned and studies are difficult to compare because they often measure repertoires differently. Studies of brain structure also indicate species differences, and open questions remain about the links between brain and vocal behavior^17,29^.

Parrots that live in companionship with humans provide a unique opportunity to study vocal learning. Although each social context is unique, parrots that live with humans are regularly exposed to human language and anthropogenic sounds. We took advantage of the existing population of captive parrots to engage the public in community science and to collect data on vocal repertoire sizes and use-patterns for a wide range of species. We constructed a survey (Appendix 1) that asked about the number of “words,” “phrases,” and human-associated mimicked “sounds” produced by companion parrots. Learning processes and opportunities among companion parrots necessarily differ from those experienced by their wild conspecifics. Companion parrots live in varied environments, interact with variable numbers of people, and hear different amounts of human-associated sound and language. All of these factors will influence mimicry, making it an imperfect proxy for vocal learning ability in wild parrots. At the same time, measuring mimicry by counting “words,” “phrases,” and “sounds” allows for standardized interspecific comparisons of vocal production ability. The data presented here offer the broadest survey to date of parrot vocal repertoires. Results provide insights into animal vocal learning, and open new avenues of investigation for researchers interested in vocal communication.

## Results

Our survey generated a final, cleaned, data set with information about vocal mimicry of “words,” “phrases,” and human-associated “sounds” by 877 individual parrots of 33 genera and 73 species (Appendix 2). We also collected data about each parrot’s species, age, sex, and social interactions with other parrots. Finally, we asked about vocal improvisation and contextual use of vocalizations (see “Methods” and Appendix 1 for more detail). Combined, these data allowed us to test for differences in vocal learning across a diverse sample of companion parrots. We present multiple analyses of how species identity, age, sex, and sociality covary with parrot vocal repertoires.

### Summary results on all parrots

We first explored repertoire sizes across all species. Our participant recruitment approach was biased towards including parrots that mimic human sounds, but 13 survey-takers reported on parrots that did not mimic humans. Those came from the following species: blue-and-yellow macaw (*Ara ararauna*), Pacific parrotlet (*Forpus coelestis*), grey parrot, Fischer’s lovebird (*Agapornis fischeri*), cockatiel (*Nymphicus hollandicus*), green-cheeked parakeet (*Pyrrhura molinae*), and budgerigar. All of these species were also represented in the data set by individuals that did mimic human sounds.

Subjects that mimicked human sounds ranged in age from 0 to 54 years (mean ± SD: 12.7 ± 10.8), with half of all individuals being 10 years old or younger. The data set included 471 putative males (53.7%), 297 putative females (33.9%), and 109 birds of unknown sex (12.4%). Four hundred and ninety eight individuals (57.0%) regularly interacted socially with other parrots, and 215 (24.7%) regularly interacted with conspecifics.

**Figure 1:**
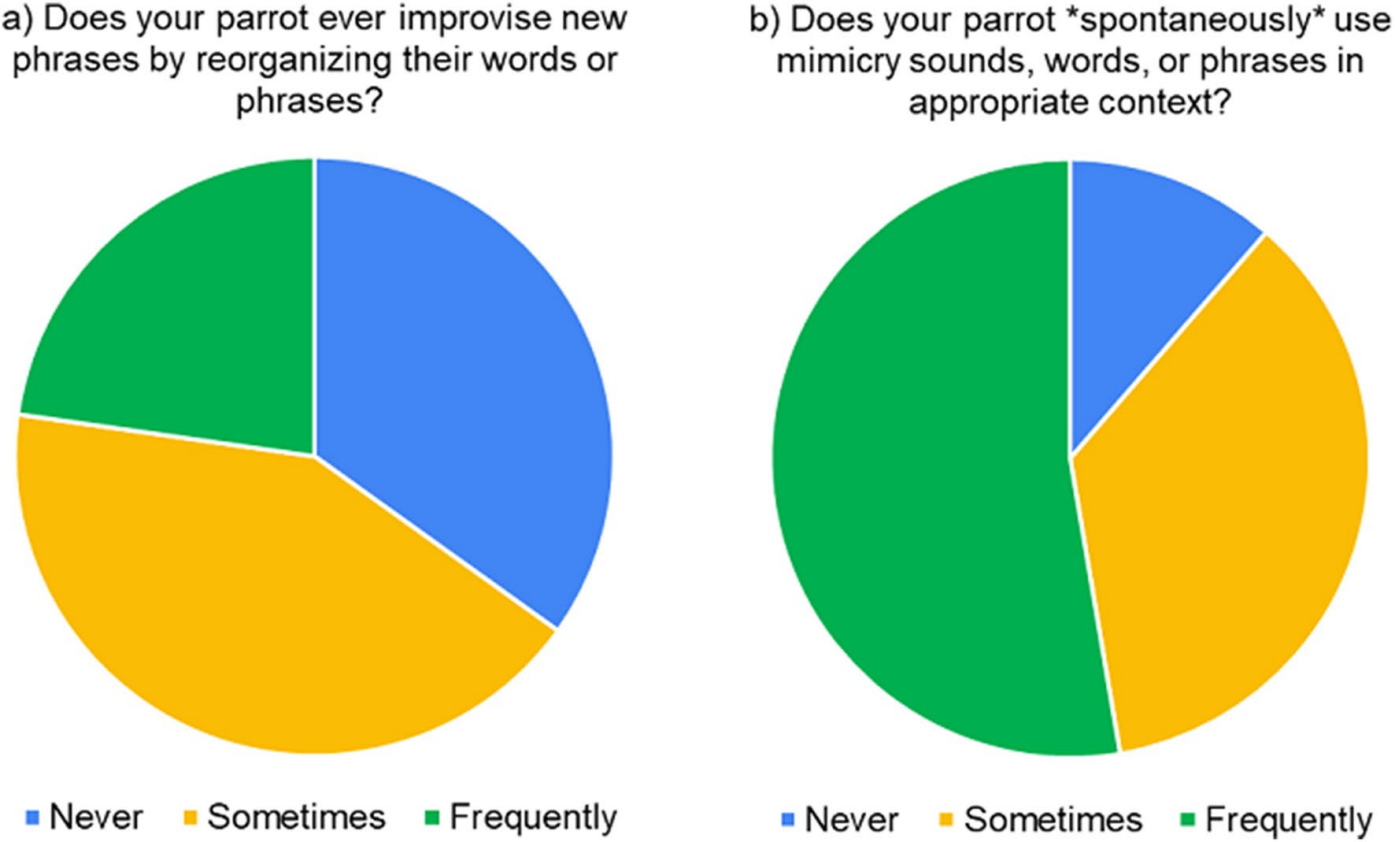
Rates of (**a**) vocal improvisation via word reorganization in phrases and (**b**) contextual use of mimicked vocalizations by companion parrots.

Mimicry repertoire sizes were highly variable, with most birds using fewer than 15 mimicked sounds, words, and phrases, while a minority of birds had repertoires of more than several hundred sounds (Table 1). Across all birds, the majority of individuals “sometimes” (42%) or “frequently” (23%) reorganized words within their vocalizations to create new phrases (Fig. 1). Survey-takers reported that most sampled parrots sometimes (36%) or frequently (53%) used their vocalizations in appropriate human contexts without human prompting (Fig. 1).

**Table 1:** Repertoire size summary statistics for all (n = 877) companion parrots reported to mimic human sounds.

Mimicry Category	Min	Quartile 1	Median	Quartile 3	Max	Mean	SD
Sounds	0	2	5	10	200	8.78	16.0
Words	0	4	11	25	550	25.5	47.2
Phrases	0	2	5	10.5	500	11.2	24.3

### Assessing biases in age and sex data

We asked survey-takers to indicate how they determined the age and sex of their parrot and used that information to classify subjects in to three confidence groups for each variable: high confidence in the age/sex data, medium confidence in the age/sex data, and low confidence in the age/sex data (see “Methods” and Appendix 3 for more detail). Birds for which we had high confidence in their age assignment had a mean age of 10.1 ± 9.6 years. Birds in the medium- and low-confidence age assignment groups had significantly higher average ages than those aged with high confidence (medium: 16.9 ± 0.7 years; low: 15.9 ± 1.1 years; ANOVA F = 37.3, p < 0.0001). Age means in the medium and low confidence groups did not differ from each other (Tukey Lower CL − 2.1, upper CL 4.0, p = 0.73).

A binomial test on all birds with sex data (unknown sex removed) revealed an overabundance of males, with a sex ratio significantly different from the expected 1:1 (61% male, Binomial test Z_768_ = − 6.24, p < 0.0001). The groups sexed with medium and low confidence also had significantly more reported males than females (medium: 71% male, Binomial test Z_121_ = − 4.55, p < 0.0001; low 74% male, Binomial test Z_180_ = − 6.48, p < 0.0001). The group sexed with high confidence did not deviate significantly from a 1:1 sex ratio (high: 54% male, Binomial Z_464_ = − 1.53, p = 0.063).

### Model tests of all species repertoire variation by age, sex and sociality

We conducted analyses of the full data set using Linear Mixed Models that accounted for uneven sampling of species by including species nested within genus as a random factor. Results of these models suggest that parrot vocal repertoires and mimicry learning do not vary consistently with sex, age, or social interactions. For a subset of 711 birds that were accurately aged as at least 2 years old, analyses found no effect of age (Table 2a). Analysis of all 768 birds with sex reported found no effect of sex (Table 2b). Linear Mixed Models on the 871 birds with social data found no effect of interacting with other parrots, whether or not they were conspecifics (Table 2c). Three models were run for each fixed factor, one per response variable. The model examining sociality data included both social interactions with any parrots and with conspecifics as fixed factors. All models met assumptions and had normally distributed residuals.

**Table 2:** Output of Linear Mixed Models testing for parrot vocal mimicry repertoire variation associated with (a) age, (b) sex, and/or (c) social interactions with other parrots.

Fixed factor	Response	Estimate	Std Err	DFDen	t	p
(a) Age	Sounds	0.00087	0.0035	655	0.25	0.80
	Words	− 0.0033	0.0044	653	− 0.75	0.46
	Phrases	− 0.0043	0.0041	613.9	− 1.05	0.30
(b) Sex	Sounds	− 0.0079	0.035	702.4	− 0.23	0.82
	Words	− 0.028	0.042	704.6	− 0.67	0.50
	Phrases	− 0.045	0.040	671.4	− 1.13	0.26
(c) Social—any parrot	Sounds	− 0.035	0.034	788.4	− 1.01	0.32
	Words	− 0.027	0.043	788.1	− 0.62	0.53
	Phrases	0.029	0.041	742.8	0.71	0.48
Social—conspecifics	Sounds	0.046	0.040	791.1	1.14	0.25
	Words	0.049	0.050	788.3	0.97	0.33
	Phrases	0.023	0.048	743.8	0.47	0.64

Because age and sex might have been assigned incorrectly for some individuals, we re-ran the LMMs using only the individuals for which we had high confidence in age (n = 400) and sex (n = 464) assignments. Results parallel those for the full data set; age and sex did not predict repertoire size (Full model output in Appendix 4).

### Species and sex differences in repertoire sizes among well-sampled species

**Figure 2:**
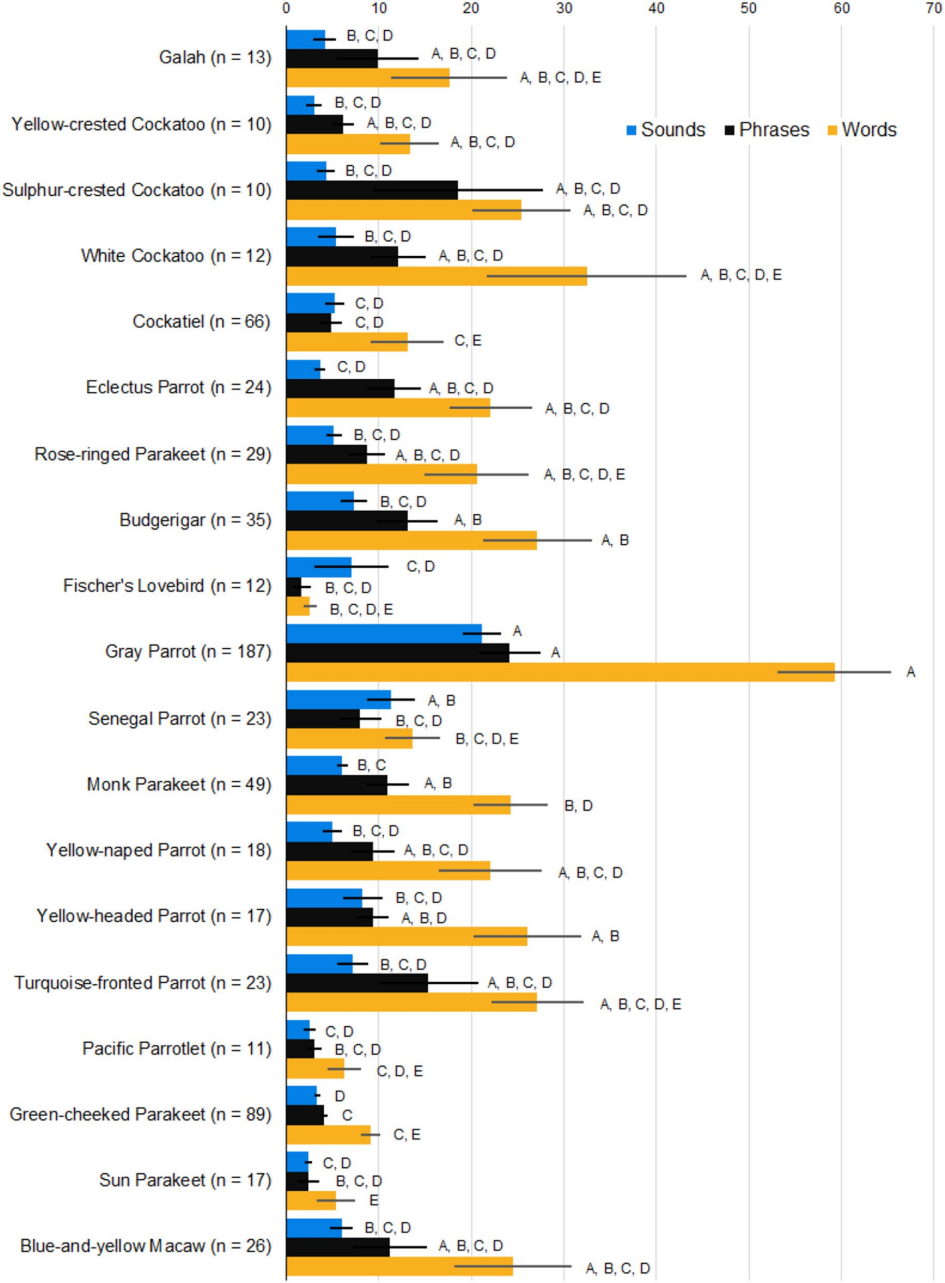
Mean (± SE) human mimicry sound, phrase, and word repertoires for 19 species of parrots. Letters following each bar indicate Tukey test statistical groupings—for each vocalization category, species not connected by a shared letter have significantly different repertoire sizes.

Nineteen species of parrots were represented by at least 10 individuals in our data set. Among those species, there were significant differences in repertoire sizes for mimicry sounds (ANOVA F_18_ = 19.37, p < 0.0001), words (ANOVA F_18_ = 10.84, p < 0.0001), and phrases (ANOVA F_18_ = 8.53, p < 0.0001) (Fig. 2), but no one species was significantly different from all others in repertoire size for any of our three mimicry categories (Fig. 2).

**Figure 3:**
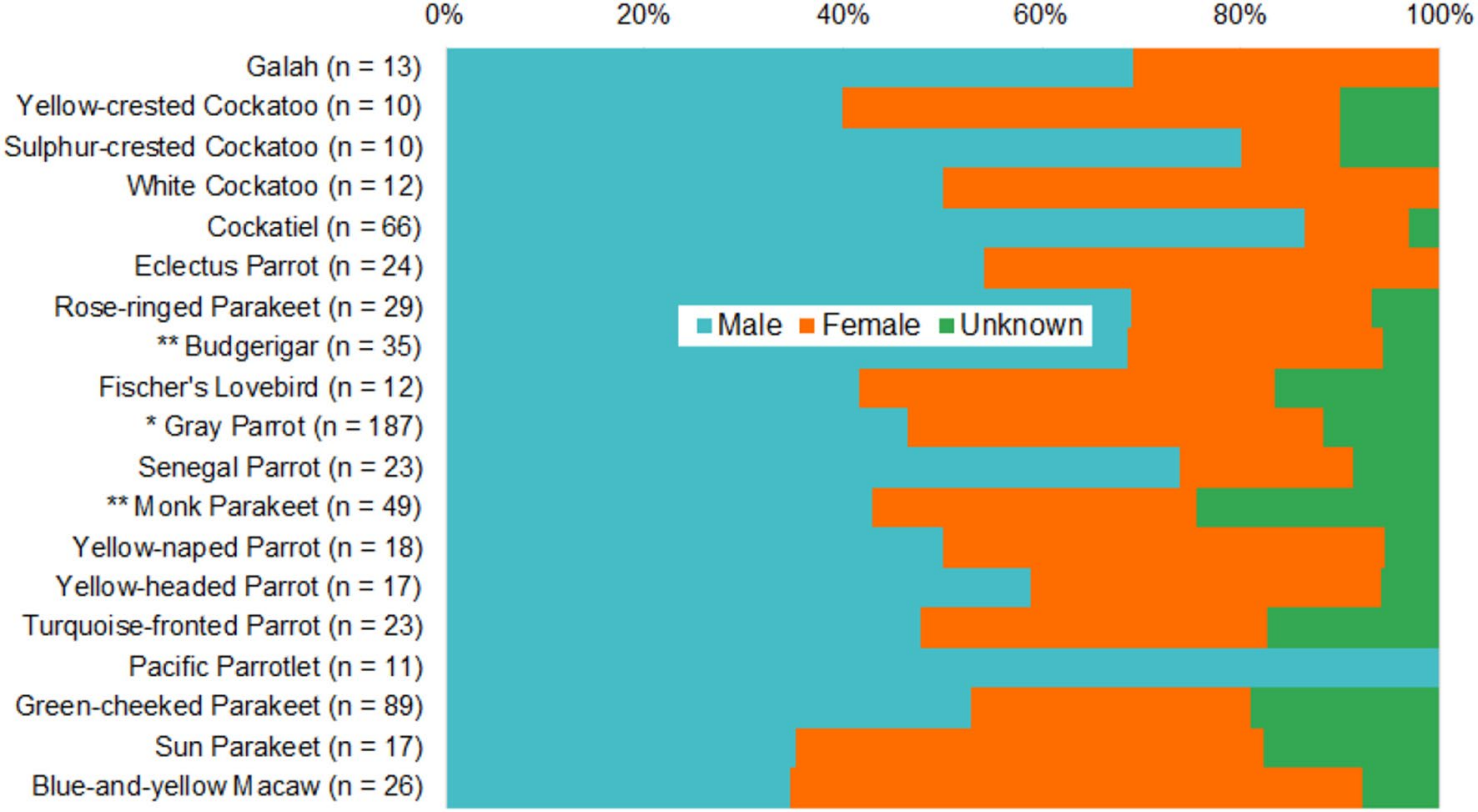
Sampling by sex of the 19 species with ≥ 10 individuals in the data set. Asterisks indicates species in which males are significantly overrepresented among birds sexed with low or medium confidence, relative to the group sexed with high confidence (Chi-squared *p < 0.05, **p < 0.01).

As in the full data set, males were overrepresented in many of our well-sampled species (Fig. 3). This might reflect a tendency for males to mimic humans more than females do, or it might reflect mistaken sex assignments. To assess the likelihood of the latter, we evaluated whether sex assignment ratios differed between the “high confidence sex-assignment” and “low/medium confidence sex-assignment” groups for each species (see Supplement). Three species had significantly more males in the low/medium confidence sex-assignment group than in the high confidence sex-assignment group: budgerigars (Chi-squared: χ^2^ = 15.71, p < 0.0001), grey parrots (Chi-squared: χ^2^ = 5.43, p = 0.020), and monk parakeets (*Myiopsitta monachus*) (Chi-squared: χ^2^ = 10.08, p = 0.0015) (Table 3).

**Table 3:** Results of linear regressions testing for associations between age and repertoire size in juvenile and adult grey parrots. Repertoires increase with age in young birds, but not in adults.

Vocalization	Juveniles aged 0–4 years (n = 25)			Adults aged 5 + years (n = 154)		
	r^2^	F	p	r^2^	F	p
Sounds	0.25	7.84	0.010*	0.0023	0.36	0.55
Words	0.45	19.74	0.0002**	0.0007	0.11	0.74
Phrases	0.38	14.41	0.0009**	0.013	2.043	0.15

*indicates that regression lines have a slope significantly different from 0 (*p < 0.05, **p < 0.01).

In 13 of the 19 well-sampled species examined, Wilcoxon rank-sum tests indicated no differences between males and females in the number of human sounds, words, and phrases that they mimicked. In one species, Pacific parrotlets, we only had data for males. In the remaining five species, we saw some evidence of sex-differences (complete data and statistics in Appendix 5). Galah (*Eolophus roseicapilla*) males had larger repertoires than females in all three vocalization categories (Sounds: Z = − 2.43, p = 0.015; Words: Z = − 2.25, P = 0.024; Phrases: Z = − 2.26, p = 0.024). Rose-ringed parakeet (*Psittacula krameri*) males had larger phrase repertoires than females (Z = − 2.26, p = 0.024). Budgerigar males had larger word (Z = − 2.03, p = 0.043) and phrase (Z = − 2.09, p = 0.036) repertoires than females. Budgerigars were one of the species in which males were overrepresented in the low and medium sex confidence groups, but we were not able to test for repertoire size differences in just the group sexed with high confidence because all individuals in that group were female. Blue and yellow macaw males had larger sound (Z = 2.89, p = 0.0039) and word (Z = 2.63, p = 0.0085) repertoires than females. Yellow-headed parrots (*Amazona oratrix*) were the only species in which females learned more from humans than males did; female yellow-headed parrots had larger mimicry sound repertoires (Z = 2.46, p = 0.014). After correction for multiple (54) comparisons, none of the above statistical results are significant. Nevertheless, we offer them as starting places for researchers wishing to look for sex differences in parrot vocal learning.

The 19 well-sampled species varied significantly in rates of word order improvisation (Chi-squared: χ^2^ = 104.7, p < 0.0001) and contextual use of vocalizations (Chi-squared: χ^2^ = 104.95, p < 0.0001) (Appendix 6). Species most likely to “frequently” rearrange vocal elements included sulphur-crested cockatoos (*Cacatua galerita*) (50% of responses), and budgerigars (39%). Species with many individuals who either “sometimes” or “frequently” rearranged vocal elements included sulphur-crested cockatoos (90%), grey parrots (85%), yellow-headed parrots (82%), turquoise-fronted parrots (*Amazona aestiva*) (82%), and yellow-naped parrots (*Amazona auropalliata*) (72%). Species most often reported to “never” rearrange vocal elements were sun parakeets (*Aratinga solstitialis*) (79%), Senegal parrots (*Poicephalus senegalus*) (52%), green-cheeked parakeets (*Pyrrhura molinae*) (52%), and Fischer’s lovebirds (*Agapornis fischeri*) (50%). Species most often reported to “frequently” use sounds, words, or phrases in appropriate context included yellow-headed parrots (76%), sulphur-crested cockatoos (75%), and grey parrots (72%). In most of the species examined, over 80% of individual parrots either “sometimes” or “frequently” used mimicry in appropriate human contexts. The only exceptions to this were rose-ringed parakeets (32% of which never used mimicry in context), sun parakeets (24% of which never used mimicry in context), and cockatiels (*Nymphicus hollandicus*) (23% of which never used mimicry in context). Frequencies of these vocal behaviors for all well-sampled species are detailed in Appendix 6.

### Focused analyses of grey parrots

Grey parrots were by far our best-sampled species, with 187 individuals represented in the data set. As reported above, grey parrots had, on average, the largest vocal mimicry repertoires for all three vocalization categories. In our tests for age effects, grey parrot repertoire sizes increased with age among juvenile birds ages 0–4, but showed no change with age in birds ages 5 and older (Table 3, Appendix 7).

Male and female grey parrots did not differ in repertoire sizes; this is true for the sample including all birds (Appendix 5) or when including only birds sexed with high-confidence (words: t_107_ = − 0.11, p = 0.91; phrases: t_104_ = − 1.00, p = 0.32; sounds: t_109_ = − 0.0008, p = 0.99). Like sex, social interactions (either with any other parrots or just with conspecifics) did not predict grey parrot repertoire variation (Table 4).

**Table 4:** Mean repertoire sizes (± SD) and results of t-tests comparing repertoire sizes in grey parrots that do and do not interact socially with other parrots.

	Alone	Social with any parrot(s)				Social with conspecific(s)			
	Repertoire	Repertoire	test n	t	p	Repertoire	test n	t	p
Sounds	16.5 ± 13.4	24.1 ± 34.8	184	1.20	0.23	18.9 ± 23.6	184	0.59	0.56
Words	56.4 ± 76.1	58.4 ± 84.4	182	0.32	0.75	60.1 ± 74.1	182	0.26	0.79
Phrases	27.5 ± 62.9	21.8 ± 27.9	178	− 0.44	0.66	24.4 ± 33.3	178	0.17	0.86

## Discussion

It is well known that parrots are excellent vocal learners; here we quantified that ability across a wide variety of species, using human mimicry as a proxy for vocal learning of natural repertoires. Results confirm that parrot vocal mimicry varies substantially both within and among species^22^. Parrot age, social interactions, and sex do not appear to be universal drivers of vocal learning ability within the order Psittaciformes, but all of these factors may have effects within individual species.

### Vocal learning variation by species

Within species, mimicry sound repertoires are extremely variable bird to bird; for example, our data indicate that a grey parrot may mimic anywhere from 0 to 600 different human words. Many other species showed smaller repertoires but similar variability. It is not entirely clear whether this range of variation would be present in natural sounds within wild parrot populations, but research has demonstrated intraspecific repertoire size variation in multiple species of parrots^30,31^.

The vast majority of parrots presented a pattern in which their repertoire size was largest for words, intermediate for phrases (composed of the reported words), and smallest for non-linguistic sounds (Fig. 2). In the wild, parrots mimic the most socially relevant vocalizations, and presumably do so in captivity as well^15^. Thus, the spoken word and phrase interactions with their human “flock” likely reflect the most socially relevant cues. The interesting exceptions to this pattern were Fischer’s lovebirds, cockatiels, and Senegal parrots who all used more sounds than phrases. Cockatiels are well-known in the pet world to be excellent whistlers, and thus it was satisfying to see that our data support that informal information. We suspect that deviations from the typical patterns may represent acoustic learning preferences, templates, or limitations^32^.

Although individual variation was substantial, we nevertheless saw strong evidence that overall vocal learning abilities differed by species. Pacific parrotlets and sun parakeets showed very limited human mimicry, while grey parrots, *Amazona* parrots, cockatoos, and macaws were generally very accomplished mimics. The patterns that we documented appeas to reflect natural vocal repertoire variation across species. The documented calls of wild parrots generally range from 5 to 15 calls^25,33–36^. Several species, however, present additional complexity: yellow-naped parrots (*Amazona auropalliata*), palm cockatoos (*Probosciger aterrimus*), and grey parrots all have natural repertoires of more than 25 discrete elements, with additional elements given in duets^13,27,37^ Members of these three groups, grey parrots, *Amazona* parrots and cockatoos also had relatively large repertoires in our study. In several of these species (particularly grey parrots) our measure of mimicked “words” (60) was higher than estimates of natural call “elements” (39) in the literature^27^. This discrepancy suggests that parrots are capable of learning vocalizations with more than 25 elements and, simultaneously, might reflect a sampling bias wherein survey-takers are more likely to report on individuals with high mimicry ability.

Parrot species varied in their tendency to improvise new combinations of elements, although most species did rearrange words to some degree. Research shows that parrot vocalization length and structure carry signal content, so there may be selective pressures favoring this ability^24,33^. If so, then our data suggest that those pressures are strongest in some cockatoos and weakest in sun parakeets and green-cheeked parakeets. In general, species with larger repertoires also showed more vocal flexibility (Fig. 2, Appendix 6). Additionally, wild birds typically use particular vocalizations in set contexts, so the ability to do so is likely to be adaptive^24^. Previous studies of captive parrots have demonstrated contextual use of mimicked words, both in tutored lab settings and in home-raised birds^28,38^. In our sample, contextual use of learned sounds was supported across 89% of individuals and most species. Survey-taker responses on this topic are necessarily subjective, so we emphasize that this rate of contextual use should be interpreted as a general estimate. Nevertheless, the data indicated that parrots frequently associated mimicked human sounds with appropriate human contexts. This finding is particularly revealing because the relevant human contexts are, by their nature, outside the range of typical wild parrot experiences. Contextual vocalization use must, therefore, rely on extremely flexible vocal learning mechanisms.

### Vocal learning variation by age

On average, birds aged with high confidence were younger than those aged with low or medium confidence. This pattern might indicate that people tend to overestimate the age of captive birds of uncertain age. This pattern might also reflect the facts that older birds are more likely to be wild-caught and that younger birds are more likely to have good hatch-date documentation. In either case, there are few ramifications of inaccurate age estimates relating to vocal behavior because our data gave no evidence that adult vocal mimicry repertoires varied with age. Our analyses of grey parrots confirmed that repertoires expanded through the juvenile phase, but did not show reliable expansion among adults. Studies of wild birds indicate that parrots can learn vocalizations throughout life; such open-ended learning is limited to a subset of vocal learning species, and can generate different outcomes as animals age^15^. In some species, animals can add new vocal features over the course of a lifetime, leading to repertoire expansion^39,40^. In other species, animals may replace parts of their repertoire with newly-learned vocalizations, leading to stable vocal production repertoire sizes across age groups^39,41^. Our data suggest that parrots fit the second pattern; although they are open-ended vocal learners, their adult repertoires change more by element replacement, than by expansion. This does not necessarily imply that vocalizations are “forgotten” through time, but merely that some sounds are no longer used as conditions change^42^. Many parrot vocalizations function in social coordination with flock-mates^22^. The fission–fusion nature of parrot flocks creates changing social conditions for each individual over its lifetime^43^. A vocal replacement model for repertoire learning would allow individuals to adjust their vocal signatures to match new social situations and stop producing vocalizations that are no longer socially relevant^11,44^.

### Vocal learning variation by sex

Our analyses of the full data set confirmed the generally held understanding that males and females in most species of parrots have similar vocal learning abilities^15^. We did, however see sex differences in some species that merit future study. First, we found a substantial overrepresentation of males in our sample. This could be interpreted several ways; (1) there are legitimately more males in the parrot pet trade, (2) pet owners are giving us accurate data but are more likely to give us data on males or (3) some bias exists in which pet owners assume their talking parrots are males, rather than females. Possibilities 1 and 2 seem unlikely because after we eliminated all parrots sexed with low confidence, we were left with a nearly 1:1 ratio of males:females in the subset of parrots that were sexed with high confidence. That trend suggests that the male bias in our data comes (at least in part) from a human tendency to label their pet parrots as male when the sex is not clear. Among songbirds, there is a strong tendency to assume that singing birds are male, and a similar bias may hold true for parrots^45^. It is unclear whether parrots in this study were mislabeled as male because they vocalize or, more simply, because that is the default human tendency for any animal.

Although we conclude that some of the male bias in our data is human error, we also saw patterns that suggest real sex differences in vocal learning some species. For example, Pacific parrotlets are a dimorphic species, and all of our sampled birds were sexed by plumage^46^. Thus, we expect sexing in this species to be fairly accurate. Our data set included 10 males and no females, a bias unlikely to result purely from sampling error. We saw a similar trend in cockatiels for which there was a large overabundance of males in the data set, even among the 17 birds sexed with high confidence. Humans may be more likely to report on parrots that are good mimics. Therefore, the results likely reflect a real-world tendency for male cockatiels to mimic more human sounds than females. Figure 3 suggests that the same might be true for galahs, sulphur-crested cockatoos, rose-ringed parakeets, Senegal parrots, and budgerigars. Existing research supports the idea that sex differences in vocal behavior are important in several of these species. Among galahs, male and female calls evoke different responses^47^, and patterns of call adjustment vary by sex among budgerigars^20^. We also note that several of these species (Pacific parrotlets, rose-ringed parakeets, budgerigars, and cockatiels; Appendix 2b) show sex-based differences in both plumage and vocal learning, raising questions about whether those traits co-evolve.

In addition to sex-based differences in the tendency to mimic humans, several well-sampled species showed evidence of sex-based differences in repertoire sizes. Particularly interesting are the blue-and-yellow macaws, in which repertoire size was significantly male-biased. We had more females (15) than males (9) in the data set, but males used on average 3–4 times as many mimicry sounds, phrases and words as females did. Galahs and budgerigars showed a similar male-bias in repertoire sizes, matching the trend of males being overrepresented in our data set for those two species. Prior research on galahs and budgerigars has found that males can be more vocal and more flexible with their vocalizations; perhaps these abilities translate to learning more call types^20,47^. A similar, but weaker, male mimicry increase occurred in rose-ringed parakeets. In only one species, yellow-headed parrots, did females show a significantly larger mimicry repertoire than males in any category (Appendix 5). Interestingly, the tendency to mimic humans (measured as sampling in the data set) and repertoire sizes did not always show the same patterns. Among sulphur-crested cockatoos, cockatiels, and Senegal parrots, males were more likely to show human mimicry, but their repertoires were not larger than the repertoires of females. This suggests that in some species, females may be less likely to mimic vocalizations, but when they do so they have just as large a vocabulary as males.

The reported sex differences in parrot vocal mimicry repertoires are intriguing, but also are tentative conclusions. In many species, including our best sampled species, grey parrots, we saw no evidence of sex-differences in repertoire size. The sex-biases that we did document lose statistical significance after controlling for the many comparisons that we conducted. Nevertheless, we expect that some of our data represent true biological differences, especially because studies of wild birds have shown similar trends^47,48^. Thus, we offer our data as a starting point for additional research. Taken together, the analyses by sex provide interesting points of comparison to other vocal learning animals. Our combined analyses suggest that sex differences in vocal learning are vastly smaller and less common among parrots than they are among oscine passerines and hummingbirds^45,49,50^. Sex-based patterns of vocal learning in parrots appear more similar to those of vocal learning mammals than to those of other vocal learning birds^51^. Overall, parrots and songbirds present excellent comparative study systems for all aspects of sex differences in song learning, from the mechanistic to the functional^17,51^.

### Vocal learning variation by social context

Many parrot vocalizations function in social organization for individuals within flocks, and the ability to learn from conspecifics is essential to parrot familial and social integration^12,15,52^. Although our study specifically examined vocal learning of human sounds, we thought it possible that the presence of other parrots would increase mimicry rates if parrots learned human vocalizations from their parrot companions. Anecdotal stories of parrots teaching words to other parrots abound^53^, and studies of grey parrot cognition show that vocal modeling by multiple tutors can lead to better learning of human words^54^. Most existing results, however, are based on human tutoring, with controlled studies of parrot-parrot word transmission lacking. Here we tested whether social interactions with other parrots correlated with more vocal learning of human sounds. Our data gave no evidence that parrot-parrot social interactions drive human vocal mimicry. This was true across the full sample (controlling for species identity), and for our best sampled species, grey parrots. Although companion parrots are known to learn from conspecifics, that learning does not appear to shape repertoire sizes^53^. Open questions remain about whether signal complexity, repertoire size, or aspects of vocal learning covary with social complexity at a larger scale among parrots^55^. Follow up studies should address these questions using phylogenetically-controlled methods^56^.

## Conclusions

Vocal learning allows for flexible, information-rich animal communication signals, and appears to have co-evolved among parrots with other specialized behavioral abilities, such as rhythmic entrainment and emotional contagion^57,58^. Evaluating vocal abilities thus allows for a better understanding of multiple components of animal sociality, learning, and behavior. Here we present evidence of vocal learning variation both within and between multiple species of parrots. We show that parrot vocal learning is generally similar in males and females, but that some species have evolved sex differences. Parrots learn new vocalizations throughout life, but do not show strong patterns of ontogenetic expansion in repertoire sizes among adults. Overall, our data make use of public contributions to advance our understanding of animal vocal learning. Although our community science data lack the precision of intensive lab and field studies, they provide a valuable comparative resource for animal communication researchers. Future work could pair the data reported here with information on parrot neuroanatomy and ontogeny to develop this group as a model for vocal production learning, a critical component of spoken language. Studies of vocal learning in animals and humans have many exciting questions yet to explore^59^.

## Methods

### Data collection

We constructed a survey (full text in Appendix 1) using Google forms (https://www.google.com/forms/about/) and collected responses between October 5th, 2020 and April 18th 2021. We recruited survey takers via email and social media with help from parrot advocacy, parrot care, and parrot interest groups. All methods were carried out in accordance with relevant guidelines and regulations. All survey-takers were informed that survey participation was entirely voluntary. Survey takers reported on one parrot each time they filled out the form. The survey asked for the name and email of the person filling out the form, and then asked 17 questions about the parrot, including information relating to species, sex, age, and vocal repertoire. We separated vocal repertoires into three categories and asked survey takers how many mimicked “sounds,” “words,” and “phrases” each parrot used. Avian vocal repertoires are commonly classified by syllable type and song type; here we included “words” as a category analog to syllable types, and “phrases” as a category analog to song types^5^. Mimicked sounds might include a single syllable, such as the simple “woof” of a dog’s bark, or they might include multiple syllables, as in a whistled tune, but all mimicked sounds were counted as a single unit distinct from words or phrases.

We received 898 survey responses. We quality-checked the data by having two people review each response and flag entries that had evident problems (i.e. unrealistic repertoire sizes > 1000, responses that included multiple birds, obvious discrepancies between answers to different questions). Flagged entries were removed from the data set, along with entries for birds that did not mimic human sounds. For the remaining data we standardized species names to genus and species following the Clements Checklist of Birds of the World^60^. Species names were left blank for hybrids or individuals with poor species data. Because age and sex can be hard to determine in some parrot species, we gave confidence values to those data for each bird. See Appendix 2 for complete confidence value rubrics for age and sex. In brief, age estimates were given high confidence if the parrot’s hatch date was known or if it was adopted as a chick. Age estimates based on behavior or adoption date were given low confidence, and those based on bands or expert diagnosis were given medium confidence (Appendix 2a). Sex assignments were marked as high confidence if sex was determined by genetic testing or egg-laying. Sex determinations based on behavior, vocalizations, or a lack of egg-laying were considered low confidence. Appearance gave different sex assignment confidence values depending on the level and consistency of plumage dimorphism in a species, with some being low (e.g. *Psittacus erithacus*), medium (e.g. *Melopsittacus undulatus*), or high (e.g*. Eclectus roratus*) (Appendix 2b). We converted social information to categorical variables, indicating whether or not each parrot regularly interacted with other parrots of any species, and whether or not each parrot regularly interacted with members of its own species. Two reviewers checked all data. Most responses in the final data set included all data fields, but some were left blank by survey respondents, generating variation in sample size across our analyses of species, age, sex, and socialization variables.

### Data analysis

All statistics were performed in JMP 13.0 (https://www.jmp.com). We first generated summary statistics about our sampling by species, age, sex, and sociality. To evaluate whether age distributions were generally robust, we tested for differences in the average age of birds within our high, medium, and low age assignment confidence groups using an ANOVA and post-hoc Tukey test. We tested for differences in the sex ratios of birds that were sexed with high, medium, and low confidence; those tests were run as binomial tests with the null hypothesis of a 1:1 ratio of males to females.

To assess general mimicry and learning patterns across all members of the order Psittaciformes we ran Linear Mixed Model (LMM) analyses testing for effects of age, sex, and social interactions on the three repertoire size variables using the full data set (n = 877). All models included species nested within genus as a random factor, but we did not test for species differences in vocal learning with this approach because many of our sampled species provided limited data, having only a single data point. Instead, we tested for species effects across 19 well-sampled species, as described below. Our age-based, sex-based, and sociality-based analyses each included three models—one per repertoire category (words, phrases, and sounds). The age-based models included age as a fixed factor and removed birds under two years of age in order to focus on learning by adult birds. The sex-based models included sex as a fixed factor and removed all birds of “unknown” sex. The sociality analyses include two fixed factors: regular social interaction with any parrots, and regular social interaction with conspecifics. All LMMs were conducted using a Standard Least Squares model and Restricted Maximum Likelihood. We log transformed the response variables (repertoire sizes) for normality, and tested for good model fits by plotting and examining residuals for normality. We did not run phylogenetically-controlled models because we focus on presenting summary data describing species traits, rather than correlating traits in an evolutionary context.

Following the order-wide analyses, we looked closely at a subset of 19 species for which we had more than 10 individuals in the data set (n = 671). We searched for evidence of mistaken sex assignments in some species by comparing the proportion of males in the high-confidence sex assignment group to the proportion of males in the combined medium- and low-confidence sex assignment groups. We calculated summary statistics for all three categories of repertoires by species and for males and females of each species. We tested for species differences in repertoire sizes using ANOVAs and post-hoc Tukey tests. We tested for species-based sex differences in vocal repertoires using Wilcoxon rank-sum tests per species. We report summary statistics on rates of vocal improvisation and contextual vocalization use by species identity; we tested for species differences in these two traits with a contingency table chi-squared analysis. Finally, we assessed age, sex, and social interactions as potential drivers of vocal repertoire sizes in our best sampled species: grey parrots. We used linear regression to look for evidence of repertoire expansion with age in both juveniles (ages 0–4) and adults (age 5 +). We used a contingency table chi-squared analysis to test whether sex ratios differed between birds sexed with high confidence, and those sexed with medium or low confidence. We assessed potential repertoire size differences by sex and social housing condition using *t* tests.

## Supplementary Information

Supplementary Information.

## Data Availability

The data set used for all analyses reported here is publicly available at this link: https://figshare.com/articles/dataset/Data_from_What_Does_Polly_Say_A_Survey_of_Vocal_Mimicry_in_Companion_Parrots/19674330.

## References

[1] Beecher MD (2021). Why are no animal communication systems simple languages?. Front. Psychol..

[2] Berwick RC, Friederici AD, Chomsky N, Bolhuis JJ (2013). Evolution, brain, and the nature of language. Trends Cogn. Sci..

[3] Nowicki S, Searcy WA (2014). The evolution of vocal learning. Curr. Opin. Neurobiol..

[4] Janik VM, Knörnschild M (2021). Vocal production learning in mammals revisited. Philos. Trans. R. Soc. B Biol. Sci..

[5] Jarvis E (2019). Evolution of vocal learning and spoken language. Science.

[6] Catchpole CK, Slater PJB (2003). Bird Song: Biological Themes and Variations.

[7] Naguib M, Janik V, Clayton N, Zuberbuhler K (2009). Vocal Communication in Birds and Mammals.

[8] ten Cate C (2021). Re-evaluating vocal production learning in non-oscine birds. Philos. Trans. R. Soc. B Biol. Sci..

[9] Pepperberg IM (2010). Vocal learning in Grey parrots: A brief review of perception, production, and cross-species comparisons. Brain Lang..

[10] Baker MC (2003). Local similarity and geographic differences in a contact call of the Galah (*Cacatua roseicapilla assimilis*) in Western Australia. Emu.

[11] Balsby TJS, Momberg JV, Dabelsteen T (2012). Vocal imitation in parrots allows addressing of specific individuals in a dynamic communication network. PLoS ONE.

[12] Berg KS, Delgado S, Cortopassi KA, Beissinger SR, Bradbury JW (2012). Vertical transmission of learned signatures in a wild parrot. Proc. R. Soc. B Biol. Sci..

[13] Dahlin C, Wright T (2007). Pair duets in the yellow-naped amazon (*Amazona auropalliata*): Phonology and syntax. Behaviour.

[14] Wright TF (1996). Regional dialects in the contact call of a parrot. Proc. R. Soc. Lond. B Biol. Sci..

[15] Bradbury JW, Balsby TJS (2016). The functions of vocal learning in parrots. Behav. Ecol. Sociobiol..

[16] Beckers GJL, Nelson BS, Suthers RA (2004). Vocal-tract filtering by lingual articulation in a parrot. Curr. Biol..

[17] Chakraborty M (2015). Core and shell song systems unique to the parrot brain. PLoS ONE.

[18] Jarvis ED (2007). Neural systems for vocal learning in birds and humans: A synopsis. J. Ornithol..

[19] Walløe S, Thomsen H, Balsby TJ, Dabelsteen T (2015). Differences in short-term vocal learning in parrots, a comparative study. Behaviour.

[20] Hile AG, Plummer TK, Striedter GF (2000). Male vocal imitation produces call convergence during pair bonding in budgerigars *Melopsittacus undulatus*. Anim. Behav..

[21] Kline CA, Dooling RJ (1984). Acoustic analysis of the budgerigar vocal repertoire. J. Acoust. Soc. Am..

[22] Bradbury JW, de Waal FBM, Tyack PL (2013). Vocal communication in wild parrots. Animal Social Complexity.

[23] Krams I, Krama T, Freeberg TM, Kullberg C, Lucas JR (2012). Linking social complexity and vocal complexity: A parid perspective. Philos. Trans. R. Soc. B Biol. Sci..

[24] Montes-Medina AC, Salinas-Melgoza A, Renton K (2016). Contextual flexibility in the vocal repertoire of an Amazon parrot. Front. Zool..

[25] Saunders DA (1983). Vocal repertoire and individual vocal recognition in the short-billed white-tailed black cokcatoo *Calyptorhynchus funereus latirostris* CArnaby. Wildl. Res..

[26] Kalhagen, A. 8 Best Talking Bird Species to Keep as Pets. *The Spruce Pets* https://www.thesprucepets.com/top-talking-bird-species-390534 (2020). Accessed on 8 September 2022.

[27] May DL (2004). The Vocal Repertoire of Grey Parrots (Psittacus erithacus) Living in the Congo Basin.

[28] Pepperberg IM (2009). The Alex Studies: Cognitive and Communicative Abilities of Grey Parrots.

[29] Sol D (2022). Neuron numbers link innovativeness with both absolute and relative brain size in birds. Nat. Ecol. Evol..

[30] Smith-Vidaurre G, Araya-Salas M, Wright TF (2020). Individual signatures outweigh social group identity in contact calls of a communally nesting parrot. Behav. Ecol..

[31] Moravec ML, Striedter GF, Burley NT (2006). Assortative pairing based on contact call similarity in budgerigars *Melopsittacus undulatus*. Ethology.

[32] Vernes SC (2021). The multi-dimensional nature of vocal learning. Philos. Trans. R. Soc. B Biol. Sci..

[33] Balsby TJS (2017). Function of vocalization length and warble repertoire size in orange-fronted conures. Anim. Behav..

[34] Cortopassi KA, Bradbury JW (2006). Contact call diversity in wild orange-fronted parakeet pairs, *Aratinga canicularis*. Anim. Behav..

[35] Fernández-Juricic E, Martella MB, Alvarez EV (1998). Vocalizations of the blue-fronted amazon (*Amazona aestiva*) in the Chancaní Reserve, Córdoba Argentina. Wilson Bull..

[36] Rowley I (1990). Behavioural Ecology of the Galah Eolophus roseicapillus in the Wheatbelt of Western Australia.

[37] Zdenek CN, Heinsohn R, Langmore NE (2015). Vocal complexity in the palm cockatoo (*Probosciger aterrimus*). Bioacoustics.

[38] Colbert-White EN, Covington MA, Fragaszy DM (2011). Social context influences the vocalizations of a home-raised African Grey parrot (*Psittacus erithacus erithacus*). J. Comp. Psychol..

[39] Kipper S, Kiefer S, Brockmann HJ (2010). Age-related changes in birds’ singing styles: On fresh tunes and fading voices?. Advances in the Study of Behavior.

[40] Nottebohm F, Nottebohm ME (1978). Relationship between song repertoire and age in the canary *Serinus canarius*. Z. Für Tierpsychol..

[41] Araya-Salas M, Wright T (2013). Open-ended song learning in a hummingbird. Biol. Lett..

[42] Nelson DA (2000). Song overproduction, selective attrition and song dialects in the white-crowned sparrow. Anim. Behav..

[43] Hobson EA, Avery ML, Wright TF (2014). The socioecology of Monk Parakeets: Insights into parrot social complexity. Auk.

[44] Balsby TJS, Bradbury JW (2009). Vocal matching by orange-fronted conures (*Aratinga canicularis*). Behav. Process..

[45] Odom KJ, Benedict L (2018). A call to document female bird songs: Applications for diverse fields. Auk.

[46] Noble C (1985). Forpus Fanciers. AFA Watchb..

[47] Scarl JC (2009). Male and female contact calls differentially influence behaviour in a cockatoo, the Galah (*Eolophus roseicapillus*). Emu Austral Ornithol..

[48] Hile AG, Burley NT, Coopersmith CB, Foster VS, Striedter GF (2005). Effects of male vocal learning on female behavior in the Budgerigar, *Melopsittacus undulatus*. Ethology.

[49] Clark CJ, Rankin D, Johnson K (2018). Female song in Costa’s Hummingbird (Calypte costae). Wilson J. Ornithol..

[50] Price JJ (2015). Rethinking our assumptions about the evolution of bird song and other sexually dimorphic signals. Front. Ecol. Evol..

[51] Carouso-Peck S, Goldstein MH, Fitch WT (2021). The many functions of vocal learning. Philos. Trans. R. Soc. B Biol. Sci..

[52] Gilardi JD, Munn CA (1998). Patterns of activity, flocking, and habitat use in parrots of the Peruvian amazon. Condor.

[53] Franklin, A. Swearing parrots removed from public view at popular Lincolnshire attraction - Lincolnshire Live. https://www.lincolnshirelive.co.uk/news/local-news/lincolnshire-wildlife-park-swearing-parrots-4554933 (2020). Accessed on 8 September 2022.

[54] Pepperberg IM, Sandefer RM, Noel DA, Ellsworth CP (2000). Vocal learning in the grey parrot (*Psittacus erithacus*): Effects of species identity and number of trainers. J. Comp. Psychol..

[55] Marcolin F, Cardoso GC, Bento D, Reino L, Santana J (2022). Body size and sexual selection shaped the evolution of parrot calls. J. Evol. Biol..

[56] Peckre L, Kappeler PM, Fichtel C (2019). Clarifying and expanding the social complexity hypothesis for communicative complexity. Behav. Ecol. Sociobiol..

[57] Schachner A, Brady TF, Pepperberg IM, Hauser MD (2009). Spontaneous motor entrainment to music in multiple vocal mimicking species. Curr. Biol..

[58] Schwing R, Nelson XJ, Wein A, Parsons S (2017). Positive emotional contagion in a New Zealand parrot. Curr. Biol..

[59] Wright TF, Derryberry EP (2021). Defining the multidimensional phenotype: New opportunities to integrate the behavioral ecology and behavioral neuroscience of vocal learning. Neurosci. Biobehav. Rev..

[60] Clements J (2021). The Clements Checklist of Birds of the World.

