# Supplementary material for: A survey of vocal mimicry in companion parrots
Source: Sci Rep. 2022 Dec 5;12:20271. doi: 10.1038/s41598-022-24335-x (PMC9722931; doi:10.1038/s41598-022-24335-x)

A Survey of Vocal Mimicry in Companion Parrots

Lauryn Benedict[1], **Alexandra Charles**[1], **Amirah Brockington**[1], **and Christine Dahlin**[2]

[1] University of Northern Colorado, School of Biological Sciences, Greeley CO, 80639, USA

[2] University of Pittsburgh at Johnstown, Department of Biology, Johnstown, PA, 15904, USA

*lauryn.benedict@unco.edu

Supplementary Information

Supplementary materials include:

- Appendix 1 - Survey text
- Appendix 2 - Sampling by species
- Appendix 3 – Sex (a) and Age (b) assignment confidence rubrics
- Appendix 4 – Output from models testing for age and sex effects among birds with high confidence age and sex assignments
- Appendix 5 - Repertoire size by sex for 19 well-sampled species
- Appendix 6 - Rates of improvisation and contextual vocalization use among well-sampled species
- Appendix 7 – Mimicked sound, phrase, and word repertoire sizes for grey parrots aged a) 0-4.5 and b) 5-50.

Appendix 1 - Survey text

Survey Link:
https://docs.google.com/forms/d/e/1FAIpQLSfMjo_mgtPpI3GHv3w90l64CaPWuPAJZWrDv3kF0q_lff1LBQ/closedform

Header: What Does Polly Say?

Birds have amazing abilities to learn new vocalizations and communication signals. Parrots that live in companionship with humans provide a unique opportunity to gather data about what birds of different species, ages, and sexes can learn. If you live or work with a captive parrot, we need your help! By filling out the survey below you will be contributing to research on parrot vocal behavior.

The survey will ask you 17 questions about your parrot. Parrots include: parakeets, amazons, macaws, cockatoos, cockatiels, lovebirds, and any other members of the order Psittaciformes. In particular, we will ask about three types of mimicked sounds that your parrot might make. You do not need to report on any natural "parrot sounds" - we are interested only in sounds that parrots have learned from living with humans.

1) A "mimicry sound" is a set of sounds always made together, in sequence, that mimics something in the parrot's environment, but does not consist of words. This category would include sounds like a dog's bark, a doorbell, or a whistled tune from a recognizable song.

2) A "phrase" is a set of words frequently and consistently said together "Polly wants a cracker" and "Polly is a nice bird" would each be a phrase.

3) A "word" is a single word. The phrase "Polly wants a cracker" includes 4 words. If your parrot regularly says "Polly wants a cracker" and "Polly is a nice bird" that is 7 words - do not count "Polly" or "a" twice when counting words.

Before you begin the survey, please attempt to count the number of mimicry sounds, phrases, and words that your bird makes as accurately as possible. Once you have that information ready, the survey should take about 10 minutes to complete. If you have multiple parrots to report on, please fill out the survey multiple times; once for each parrot.

Survey participation is entirely voluntary. If you begin the survey you may decide to stop and withdraw at any time. Data collected here will be used in academic research publications. All names and email addresses will be kept strictly confidential.

About us: this research is being conducted by Alexandra Charles, Dr. Lauryn Benedict, and Dr. Christine Dahlin. We are a student and two professional ornithologists with expertise in bird vocalizations and Parrot Biology. Read more about Lauryn Benedict here: https://www.unco.edu/nhs/biology/about-

us/benedict-lauryn/
Read more about Christine Dahlin here: https://christinedahlin.weebly.com/.

Contact us at the following email address: WhatDoesPollySay@gmail.com.
Email*
Valid email required

Please provide your name:

- About your parrot -

In this section we will collect basic information about your parrot

1. Please provide your parrot's name:
2. What species is your parrot?
3. Is your parrot male or female? (choices: Male, Female, Unknown)
4. How did you determine your parrot's sex? Check all that apply. (Choices: Appearance, Genetic Testing, Behavior, Egg-Laying, Veterinary/Expert Diagnosis, Other - write in)
5. How old is your parrot in years? If you don't know, leave the answer space blank.
6. How did you determine your parrot's age?
7. Does your parrot mimic human speech and/or other sounds (doorbells, barking dogs. etc.)? (Options: Yes, No)

- Parrot bonding -

The next three questions are about your parrot's social environment. If you answer 'no' to the next question, please skip forward to the 'Parrot Vocal Behavior' section and question 11.

Reminder: the "focal parrot" is the parrot that you are focusing on when answering the questions of this survey. If you have more than one parrot, please repeat this survey for the other parrots.

8. Does the focal parrot interact socially with other parrots? Social interaction could include: exchanging vocalizations, physical interactions, or sharing a cage. (Options: Yes, No)
9. How many other parrots does the focal parrot interact with socially?
10. How many of the other parrots are the same species as the focal parrot?

- Parrot vocal behavior -

Important terminology for the next questions:

Parrots make a wonderful range of sounds, including "parrot sounds" that are natural to their species,

and mimicked sounds that they have learned from humans or other species. For the purposes of this study, we will ask you to report on mimicked sounds only.

There are three specific types of mimicked sounds that we will ask about:

1) A "mimicry sound" is a set of sounds always made together, in sequence, that mimics something in their environment, but does not consist of words. This category would include sounds like a dog's bark, a doorbell, or a whistled tune from a recognizable song.

2) A "phrase" is a set of words frequently and consistently said together "Polly wants a cracker" and "Polly is a nice bird" would each be a phrase.

3) A "word" is a single word. The phrase "Polly wants a cracker" includes 4 words. If your parrot regularly says "Polly wants a cracker" and "Polly is a nice bird" that is 7 words - do not count "Polly" or "a" twice when counting words.

11. How many different non-speech "mimicry sounds" does your parrot make?
12. How many different "phrases" does your parrot use?
13. How many different "words" does your parrot use within all of their combined phrases?
14. Does your parrot ever improvise new phrases by reorganizing the words or phrases you reported on above?
Options:

- No, my parrot only uses words and phrases in set patterns that are the same every time
- Most of the time my parrot uses words and phrases in set patterns, but occasionally reorganizes words
- My parrot frequently "remixes" their words and phrases into new combinations

15. Does your parrot *spontaneously* use mimicry sounds, words, or phrases, that match the appropriate human context for that sound/phrase/word? For example, this would include making a doorbell sound when someone new enters the house or saying "feed me" just before dinnertime. This would not include mimicry such as responding "hello" to a person who said "hello".
Options:

- My parrot FREQUENTLY uses sounds/phrases/words in appropriate human context without human prompting
- My parrot SOMETIMES uses sounds/phrases/words in appropriate human context without human prompting
- My parrot NEVER uses sounds/phrases/words in appropriate human context without human prompting)

16. *Optional* Please list some of the words or phrases your parrot uses and their contexts
17. Is there anything else you would like to tell us?

Press the Submit button below to submit your responses. Thank you for taking the survey! If you know other parrots and their humans, please pass the survey on by sharing the URL.

Appendix 2 - Sampling by species of the 877 parrots included in vocal learning analyses

Genus	Species	n
Agapornis	fischeri	12
Agapornis	roseicollis	1
Amazona		3
Amazona	aestiva	23
Amazona	albifrons	1
Amazona	amazonica	3
Amazona	auropalliata	18
Amazona	autumnalis	3
Amazona	brasiliensis	4
Amazona	leucocephala	1
Amazona	ochrocephala	7
Amazona	oratrix	17
Amazona	viridigenalis	4
Ara		2
Ara	ararauna	26
Ara	chloropterus	9
Ara	glaucogularis	2
Ara	macao	6
Ara	militaris	2
Ara	severus	4
Aratinga	jandaya	2
Aratinga	nenday	2
Aratinga	solstitialis	17
Bolborhynchus	lineola	6
Brotogeris	versicolurus	2
Cacatua		1
Cacatua	alba	12
Cacatua	galerita	10
Cacatua	goffiniana	9
Cacatua	moluccensis	9
Cacatua	sanguinea	3
Cacatua	sulphurea	10
Cyanoliseus	patagonus	2
Cyanoramphus	novaezelandiae	1
Diopsittaca	nobilis	7
Eclectus	roratus	24
Eolophus	roseicapilla	13
Eos	bornea	2
Eupsittula	aurea	4
Eupsittula	canicularis	3
Forpus	coelestis	11

Lophochroa	leadbeateri	2
Lorius	garrulus	2
Melopsittacus	undulatus	35
Myiopsitta	monachus	49
Neophema	bourkii	2
Nymphicus	hollandicus	66
Pionites	caique	3
Pionites	leucogaster	5
Pionites	xanthomerius	2
Pionites	melanocephalus	4
Pionus	chalcopterus	2
Pionus	maximiliani	5
Pionus	menstruus	3
Poicephalus	fuscicollis	1
Poicephalus	gulielmi	2
Poicephalus	meyeri	5
Poicephalus	rufiventris	1
Poicephalus	senegalus	23
Polytelis	alexandrae	3
Polytelis	anthopeplus	1
Primolius	auricollis	3
Psittacara	finschi	8
Psittacara	mitratus	3
Psittacula	derbiana	2
Psittacula	krameri	29
Psittacus	erithacus	187
Pyrrhura	frontalis	3
Pyrrhura	molinae	89
Pyrrhura	perlata	4
Pyrrhura	rhodocephala	5
Pyrrhura	rupicola	3
Thectocercus	acuticaudatus	9
Touit	dilectissimus	1
Trichoglossus	chorolepidotus	1
Trichoglossus	moluccanus	6

Appendix 3 - Sex and Age assignment confidence rubrics

a) Parrot age confidence assignment rubric

High Confidence	Hatch Date<br>Breeder Knowledge<br>Juvenile Ownership
Medium Confidence	Previous Owner Knowledge<br>Leg Band<br>Veterinary/Expert Confirmation
	Appearance:<br>● Juvenile Plumage
Low Confidence	Appearance with no info<br>Behavior<br>Date of Purchase/Adoption

b) Parrot sex confidence assignment rubric:

High Confidence	Genetic Testing<br>Egg-Laying
	Appearance:<br>● Eclectus parrot (*Eclectus roratus*)
Medium Confidence	Veterinary/Expert Confirmation<br>Breeder Confirmation<br>Past Owner Confirmation
	Appearance:<br>● Pacific parrotlet (*Forpus coelestis*)<br>● Lord Derby's parakeet (*Psittacula derbiana*)<br>● Rose-ringed parakeet (*Psittacula krameri*)<br>● Budgerigar (*Melopsittacus undulatus*)<br>● Cockatiel (*Nymphicus hollandicus*)
Low Confidence	Behavior<br>Appearance<br>Word/Phrase Usage<br>Unknown<br>Non-Egg-Laying

Appendix 4 - Models testing for age and sex effects among birds with high confidence age and sex assignments

Output of Linear Mixed Models, using only birds aged and sexed with high confidence, testing for parrot vocal mimicry repertoire variation associated with age (n=400) and sex (n=474).

Fixed factor	Response	Estimate	Std Err	DFDen	t	P
a) Age	Sounds	0.0055	0.0054	366.9	1.02	0.31
	Words	-0.0018	0.0065	368.7	-0.28	0.78
	Phrases	-0.0043	0.0060	349.3	-0.71	0.48
b) Sex	Sounds	-0.025	0.043	421.8	-0.58	0.57
	Words	-0.06	0.052	433.6	-1.34	0.18
	Phrases	-0.059	0.049	413.5	-1.21	0.23

Appendix 5 - Repertoire size by sex for 19 well-sampled species

Means (SD) and Wilcoxon tests on the numbers of mimicry sounds, phrases, and words used by males and females of 19 species. See Figure 2 for sample sizes. Significant p-values are in red text.

	Mimicry sounds				Phrases				Words				larger repertoires
	Females	Males	Z	P	Females	Males	Z	P	Females	Males	Z	P	
Galah	1 (1.15)	5.56 (4.48)	-2.43	0.015	2 (0.82)	13.44 (18.39)	-2.26	0.024	4.5 (2.08)	23.44 (25.19)	-2.25	0.024	Male***
Yellow-crested Cockatoo	7.8 (8.32)	2.75 (1.26)	-1.36	0.17	15.4 (11.17)	6 (3.27)	-1.24	0.22	34.6 (32.36)	13 (7.39)	-1.24	0.22	Female
Sulphur-crested Cockatoo	1	4.62 (3.16)	-0.99	0.32	4	21.63 (32.0)	-1.17	0.24	8	28.75 (17.16)	-0.59	0.56	Male
White Cockatoo	2.33 (1.64)	3.67 (3.83)	0.25	0.81	6.83 (4.54)	5.5 (4.14)	-0.41	0.68	15.67 (14.40)	11 (6.54)	-0.32	0.75	Mixed
Cockatiel	10.86 (17.54)	4.56 (5.93)	0.9	0.37	12 (13.01)	4.07 (8.36	1.59	0.11	38.43 (71.85)	10.26 (23.93)	1.5	0.13	Female
Eclectus Parrot	2.91 (2.26)	4.31 (3.2)	-0.91	0.36	9.55 (14.61)	13.61 (13.59)	-1.25	0.21	16.27 (17.52)	27 (24.49)	-1.8	0.072	Male
Rose-ringed Parakeet	2.86 (1.77)	6.15 (5.19)	-1.59	0.11	2.71 (3.30)	11.15 (11.65)	-2.25	0.024	14.14 (27.39)	23.85 (32.51)	-1.52	0.13	Male*
Budgerigar	6.44 (10.85)	7.21 (7.01)	-1.08	0.28	5.22 (8.47)	17.13 (21.67)	-2.03	0.043	10.44 (17.29)	34.83 (38.67)	-2.09	0.036	Male**
Fischer's Lovebird	4.6 (5.32)	11.6 (21.5)	-0.11	0.92	1 (0.71)	3 (5.2)	-0.11	0.91	2.6 (2.79)	3 (3)	0	1	Male
Grey Parrot	23.15 (30.87)	21.83 (29.14)	0.57	0.57	21.99 (23.37)	21.83 (31.22)	0.9	0.37	60.26 (80.51)	61.05 (85.25)	0.67	0.5	Mixed
Senegal Parrot	7.75 (6.29)	12 (14.1)	-0.36	0.72	5.25 (6.70)	8.29 (12.01)	-0.09	0.93	11 (9.56)	12.59 (13.43)	0	1	Male
Monk Parakeet	6.13 (4.53)	6.14 (3.47)	-0.28	0.78	4.56 (2.85)	10.95 (12.28)	-1.65	0.098	10.93 (6.90)	24.10 (23.92)	-1.84	0.065	Male
Yellow-naped Parrot	10.13 (10.13)	7.22 (9.0)	0.58	0.56	11.63 (7.95)	8 (6.48)	0.88	0.38	27 (19.14)	27.22 (30.74)	0.24	0.81	Mixed
Yellow-headed Parrot	11.83 (9.2)	4.1 (2.85)	2.46	0.014	10.5 (5.75)	9.7 (5.5)	0.27	0.78	20.33 (11.48)	25.9 (18.27)	-0.71	0.48	Mixed*
Turquoise-fronted Parrot	4.63 (4.6)	6 (5.57)	-0.71	0.48	11.88 (16.17)	9 (7.87)	0	1	29 (39.65)	20.82 (18.15)	0.25	0.8	Mixed
Pacific Parrotlet	No Data	2.55 (2.21)			No Data	3 (2.53)			No Data	6.27 (5.91)			
Green-cheeked Parakeet	3.68 (4.41)	3.45 (3.13)	-0.56	0.58	3.4 (3.1)	4.85 (4.63)	-1.11	0.27	8.6 (11.89)	10 (10.09)	-1.27	0.2	Mixed
Sun Parakeet	1.63 (0.92)	2.83 (1.83)	1.07	0.28	0.88 (1.13)	3.17 (5.91)	0.28	0.78	2.88 (2.23)	7.5 (11.59)	0	1	Male
Blue-and-yellow Macaw	3.13 (2)	11.22 (8.29)	2.89	0.0039	5.13 (4.16)	23 (32.43)	1.84	0.066	13.4 (9.41)	46.67 (47.51)	2.63	0.0085	Male**

Appendix 6 - Rates of improvisation and contextual vocalization use among well-sampled species

Percentages of subjects reported to "never", "sometimes", or "frequently" A) show improvisation by rearranging words within phrases, and b) use mimicked sounds, words, or phrases in appropriate human context without human prompting.

	A) Does your parrot ever improvise by rearranging their words in phrases?				B) Does your parrot spontaneously use words in appropriate human context?			
Species	n	Never	Sometimes	Frequently	n	Never	Sometimes	Frequently
Galah	13	31%	54%	15%	13	8%	31%	62%
Yellow-crested Cockatoo	10	30%	50%	20%	10	0%	40%	60%
Sulphur-crested Cockatoo	10	10%	40%	50%	8	13%	13%	75%
White Cockatoo	12	33%	33%	33%	12	8%	25%	67%
Cockatiel	62	47%	40%	13%	64	23%	48%	28%
Eclectus Parrot	24	42%	46%	13%	24	8%	50%	42%
Rose-ringed Parakeet	27	41%	26%	33%	28	32%	21%	46%
Budgerigar	31	29%	32%	39%	33	18%	33%	48%
Fischer's Lovebird	8	50%	25%	25%	11	9%	55%	36%
Grey Parrot	185	15%	48%	37%	184	2%	26%	72%
Senegal Parrot	23	52%	26%	22%	23	13%	43%	43%
Monk Parakeet	49	29%	53%	18%	49	8%	37%	55%
Yellow-naped Parrot	18	28%	39%	33%	18	0%	44%	56%
Yellow-headed Parrot	17	18%	71%	12%	17	6%	18%	76%
Turquoise-fronted Parrot	22	18%	59%	23%	23	4%	43%	52%
Pacific Parrotlet	11	36%	55%	9%	10	10%	70%	20%
Green-cheeked Parakeet	87	52%	37%	11%	88	18%	44%	38%
Sun Parakeet	14	79%	14%	7%	17	24%	29%	47%
Blue-and-yellow Macaw	26	42%	42%	15%	26	8%	31%	62%

Appendix 7 – Mimicked sound, phrase, and word repertoire sizes for grey parrots aged a) 0-4.5 and b) 5-50.

a)

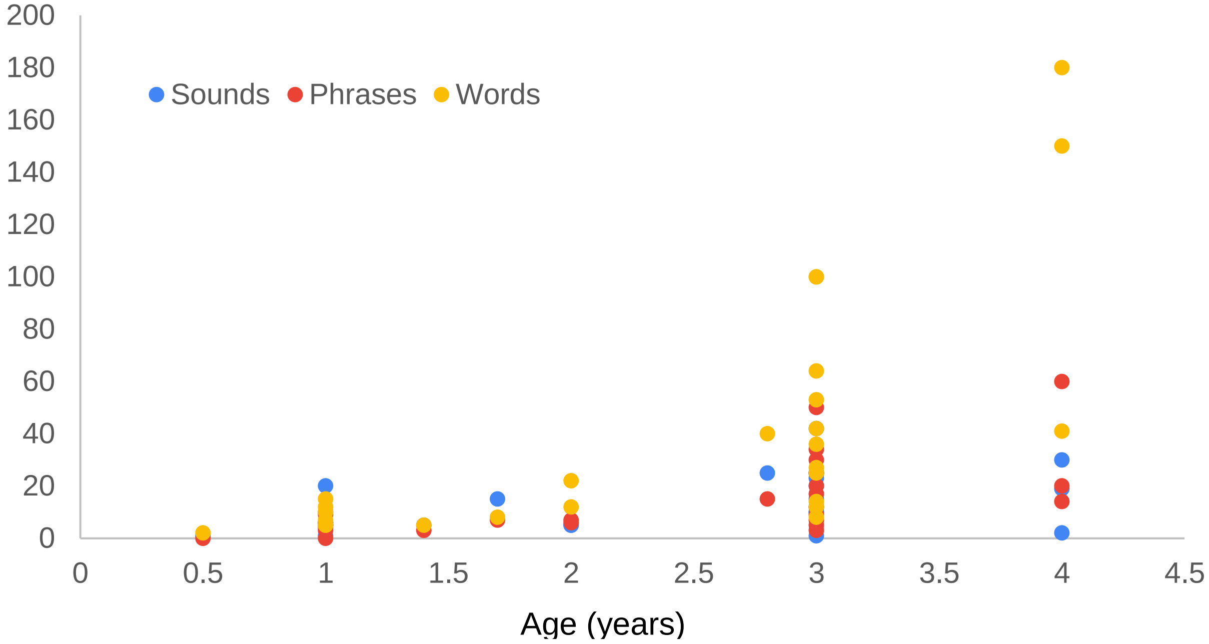

b)

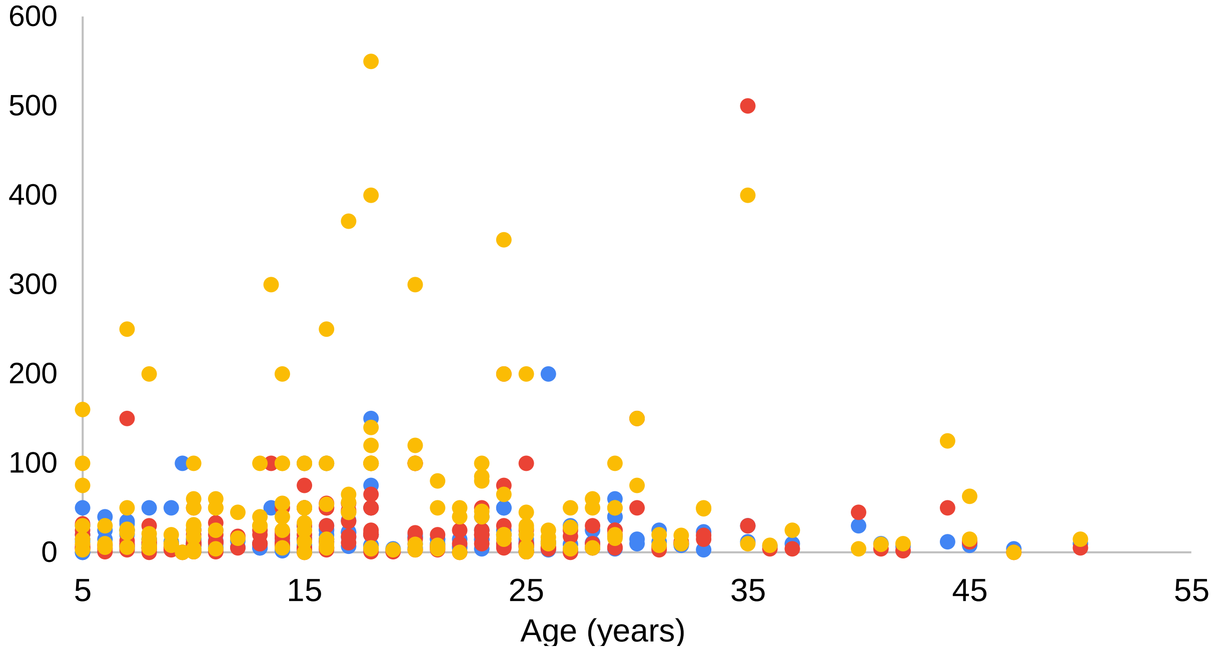

Supplement: Supplementary file 1 — Supplementary Information. [file 41598_2022_24335_MOESM1_ESM.pdf]
